# Herpes Simplex Virus Seroprevalence among Pregnant Finnish Women and Their Spouses—A Six-Year Follow-Up Cohort Study

**DOI:** 10.3390/microorganisms10081506

**Published:** 2022-07-26

**Authors:** Johanna Laakso, Tytti Vuorinen, Jaana Rautava, Katja Kero, Stina Syrjänen, Veijo Hukkanen

**Affiliations:** 1 Department of Oral Pathology and Radiology, Institute of Dentistry, and Medicity Research Laboratory, Faculty of Medicine, University of Turku, 20520 Turku, Finland; jaana.rautava@helsinki.fi (J.R.); stisyr@utu.fi (S.S.); 2 Finnish Doctoral Programme in Oral Sciences, University of Turku, 20520 Turku, Finland; 3 Institute of Biomedicine, University of Turku, 20520 Turku, Finland; tytti.vuorinen@utu.fi (T.V.); veijo.hukkanen@utu.fi (V.H.); 4 Department of Clinical Virology, Turku University Hospital, 20520 Turku, Finland; 5 Department of Oral and Maxillofacial Diseases, Clinicum, Faculty of Medicine, University of Helsinki and Helsinki University Hospital, 00290 Helsinki, Finland; 6 Department of Pathology, Medicum, Faculty of Medicine, University of Helsinki and HUS Diagnostic Center, HUSLAB, Helsinki University Hospital, 00290 Helsinki, Finland; 7 Department of Obstetrics and Gynaecology, University of Turku & Turku University Hospital, 20520 Turku, Finland; katja.kero@utu.fi; 8 Department of Pathology, Turku University Hospital, 20520 Turku, Finland

**Keywords:** herpes simplex virus, HSV, seroprevalence, pregnancy, oral sex, oral herpes

## Abstract

The aim was to evaluate the herpes simplex virus (HSV) seroprevalence and seroconversion among 285 pregnant women and their 120 male spouses in Finland during a six-year follow-up (FU) between 1998–2008. We also studied the effect of sexual habits, pregnancy, and other demographic factors on the acquisition of HSV infection. Combined HSV-1 and HSV-2-IgG antibodies were assessed in the first baseline serum samples with an indirect enzyme immunoassay method. The individuals with seronegative or borderline HSV serology at baseline were additionally tested using their latest FU serum sample available. The overall HSV seroprevalence during the FU was 58.9% (168/285) among the women and 53.3% (64/120) among their spouses. The seroconversion rate was 11.4% (15/132) and 12.5% (8/64) among women and their spouses, respectively. Both spouses were HSV seropositive in 39.2% (47/120). To determine the HSV-2 seroprevalence, we also tested all HSV-seropositive participants using HSV-2-specific antigen. HSV-2 seropositivity was detected in 10.9% (44/405) of the participants. The age (*p* = 0.006) and history of genital warts (*p* = 0.006) of the women were associated with combined HSV-1 and/or HSV-2 seropositivity, while a younger age was related to HSV seroconversion (*p* = 0.023). Among the male spouses, HSV seropositivity was associated with the practice of oral sex (*p* = 0.033). To conclude, women of childbearing age acquire primary HSV infections and the presence of HSV in oral epithelium is common among HSV-seropositive individuals.

## 1. Introduction

The herpes simplex virus type 1 (HSV-1) infection is transmitted usually via saliva and occurs most commonly in the head and neck region, while HSV-2 infects mostly the genital region and is sexually transmitted [1]. However, HSV-2 can also cause oral infections and HSV-1 genital infections [2,3]. Infection with the HSV (both HSV-1 and -2) is lifelong and in the oral region the latent HSV in the trigeminal ganglion can reactivate after various time intervals. During the active infection, HSV shedding can be detected in saliva and HSV DNA can be found in exfoliated oral mucosal cells of which gingival sulcular epithelium is the most studied [4]. Asymptomatic shedding of oral HSV-1 can happen at least monthly in ≥70% of the population or even more regularly [5]. In cross-sectional surveys, oral HSV shedding is detectable in 2–5% among adults [6].

Globally, over 70% of the adult population have antibodies to HSV [5,6,7,8]. In developing countries, HSV-1 seroconversion occurs still earlier in life, as 40–70% of adolescents have HSV-1 antibodies [7,8]. In developed countries, HSV-1 seropositivity has declined among the higher social classes during the 20th–21st centuries [8,9,10,11]. In Finland, the seropositivity for HSV-1 was 70% between the years 1988–1989 [12]. HSV-1 seroprevalence declined from 69.5 to 45% (*p* < 0.001) among pregnant Finnish women between the years 1992–2012 [11]. In the year 2000, the HSV-1 seroprevalence among 558 Finnish puerperal women was 46.8%, and 9.3% for HSV-2 [13]. In the USA, the HSV-1 seroprevalence declined by nearly 7% (*p* < 0.01) while the highest decline >29% (*p* < 0.01) was found among 14–19-year-olds from 1999–2004 to 2005–2010 [10]. HSV seroepidemiology is mostly affected by geographic location, age, socioeconomic status, personal hygiene, and behavioral factors such as age at sexual debut and number of sexual partners and is highly dependent on which kind of cohort is being studied [6,8,9,10,14].

The aim of the present study was to evaluate HSV seroprevalence and seroconversion (combined HSV-1 and HSV-2) among young pregnant women and their spouses during the six-year follow-up (FU) utilizing the Finnish Family HPV Study cohort [15,16,17,18,19,20]. Additionally, we were interested on the proportion of HSV-2 positivity among those who tested HSV seropositive. Furthermore, we also analyzed the association of HSV seropositivity in those who had previously tested HSV-1 DNA positive in their oral swab specimens as described earlier [21,22]. The association of pregnancy and demographic factors i.e., sexual habits with HSV seroprevalence, was also assessed.

## 2. Materials and Methods

### 2.1. Study Population and Clinical Samples

This study is part of the Finnish Family HPV Study which is a longitudinal cohort study of the University of Turku/Turku University Hospital, Finland, as described [15,16,17,18,19,20]. Originally, 329 families were recruited between the years 1998–2002, comprising of 329 women and their 139 male spouses and 331 neonates. An original aim of the Finnish Family HPV Study was to understand the mother–child transmission of HPV. Thus, not all of the fathers-to-be participated in the study. The inclusion criteria for a parent were baseline sampling at recruitment (36 weeks of their index pregnancy) and an informed consent to participate. This study was approved by the Research Ethics Committee of Turku University Hospital (#3/1998, #2/2006, 45/180/2010 and TO7/008/2014).

In the present sub-study, the prevalence of HSV antibodies among the women and their spouses at the entry of the study was analyzed. In total, serum samples were available from 285 women (mean age 25.5, range 18–38 years) and their 120 spouses (mean age 28.7, range 19–46 years). The HSV seroconversion during the six-year FU was assessed by selecting the last FU sample available for an additional HSV testing from all HSV-seronegative individuals and those with a borderline result at baseline. The mean timespan of the last FU sample was 57.3 (range 6.7–94.5) and 47.8 (range 5.0–91.5) months for the women and their spouses, respectively. In total, 132 women and 64 spouses were retested.

The demographic data recorded at 2 months after delivery including tobacco and alcohol use and sexual habits were available from the questionnaires as described earlier [15,16,17,18,19,20].

### 2.2. Detection of HSV-IgG Antibodies in Sera

#### 2.2.1. Blood Sample Preparation

Blood samples were taken at baseline before delivery and at 2, 6, 12, 24, 36, and 72 months after delivery as described earlier [16,17,18]. Blood samples were collected, and the serum was separated by centrifuging at 1150× *g* for 10 min (Sorvall GLC-2; DuPont Instrument). The serum was first stored at −20 °C (maximum 1 week) and thereafter at −70 °C, as previously [16,17,18].

#### 2.2.2. Indirect Enzyme Immunoassay (EIA)

Combined IgG antibodies against herpes simplex viruses (HSV-1, HSV-2) were analyzed with indirect enzyme immunoassay (EIA), as previously described [13,23]. Briefly, the antigens were purified from HSV-1- (strain F) and HSV-2 (strain G)-infected Vero cell cultures (American Type Culture Collection, Manassas, VA, USA) [13]. Polystyrene strips (Thermo) were coated with pooled HSV-1 and HSV-2 envelope antigens in phosphate buffered saline. Serum samples were tested as duplicates and incubated for 2 h at 37 °C, after which horseradish peroxidase (HRP)-conjugated anti-human-IgG (dilution 1:16,000, DAKO, Jena, Germany) was added. Then, tetramethylbenzidine was added and the reaction was stopped with 0.2 N sulfuric acid. The optical absorbance was measured at 450 nm by a BEP III analyzer (Siemens, Munich, Germany). The results were determined by comparing the average absorbance of specimens to negative (<1 EIA-units; EIU) and positive (100 EIU) controls. Sera with ≥10 EIU were scored positive, those with 5–9 EIU as borderline, and those with ≤4 EIU as negative. The seroconversion was defined as acquisition of detectable HSV-IgG antibodies.

The sera which scored positive in the HSV type-common EIA test (marked in the manuscript as HSV seropositive ones) were further studied for HSV-2 type-specific antibodies using HerpeSelect 2 ELISA IgG kit (Focus Diagnostics, Cypress, CA, USA). The assay was performed according to the manufacturer’s instructions and the results were evaluated as index values relative to the kit cut-off calibrator, as recommended by the manufacturer.

#### 2.2.3. Patient and Public Involvement

The study was based on anonymized patient records and the results were not disseminated to the participants. The participants were not involved in setting the research question, study design, study methods or in how the study was conducted.

### 2.3. Statistics

Statistical analyses were carried out using SPSS^®^ (SPSS for Windows, version 24.0.0.1, SPSS Inc., Chicago, IL, USA) and STATA (STATA/SE 14.1, StataCorp, College Station, TX, USA). Frequencies were analyzed using the χ^2^-test. Differences in the means of continuous variables were analyzed using the Mann–Whitney test or the Kruskal–Wallis test for two and multiple independent samples, respectively. ANOVA was used to derive the means (and their SD) for the continuous variables.

Oral HSV DNA status based on oral brush samples has been published earlier [21,22]. This previously published HSV DNA data was used here in the statistical analysis to disclose the possible association between the HSV serology and the presence of intraoral HSV DNA.

## 3. Results

In total, 57.3% (232/405) of the participants were HSV (combined HSV-1 and HSV-2) seropositive during the FU, the HSV seropositivity being 58.9% (168/285) in women and 53.3% (64/120) in their male spouses. At the baseline visit, 53.7% (153/285) of women and 46.7% (56/120) of their spouses were HSV seropositive. The median antibody level was significantly lower in HSV-seropositive women than in their spouses (*p* = 0.0017) (Table 1). In total, 11.4% (15/132) of the women and 12.5% (8/64) of their spouses seroconverted during FU and among the seroconverted, the HSV antibody levels were higher in women than in their spouses (*p* = 0.0007) (Table 1).

In total, 10.9% (44/405) of the participants were HSV-2 seropositive. Among the HSV-seropositive participants, 19.0% (44/232) were HSV-2 seropositive (Table 1). Among the women, the total HSV-2 seropositivity was 12.3% (35/285) and 7.5% (9/120) among the men. HSV-2 seropositivity among the HSV seropositive was 20.8% (35/168) of women and 14.1% (9/64) of men (Table 1). Of the HSV-seroconverted subjects, 21.7% (5/23) were HSV-2 positive. Four of them were women and one was a male spouse (Table 1).

In the present study, both partners in the couples were HSV seropositive in 39.2% (47/120) of the cases (Table 2). Interestingly, 87.5% (7/8) of the seroconverted men were the spouses of HSV-seropositive women. In contrast, only 26.7% (4/15) of the seroconverted women were the spouses of seropositive men. Among them, both partners of the couples were HSV-2 seropositive in 12.8% (6/47) of the cases. All couples practiced oral sex, three couples reported occasional anal sex and previous sexually transmitted infection (genital herpes, condyloma or chlamydia). Of the female spouses, 5/6 were current smokers while only one of the six spouses was a smoker. One couple had HSV-2 seroconversion detectable at the same FU visit.

The age (*p* = 0.006) and positive history of genital warts (*p* = 0.006) of the women were significantly associated with HSV seropositivity. In addition, younger age was associated with increased susceptibility to HSV seroconversion among women (*p* = 0.023) (Supplementary Table S1). Self-reported high frequency of oral sex was associated with the HSV seropositivity among the male spouses (*p* = 0.033) (Supplementary Table S2). HSV seropositivity was not associated with education, number of sexual partners, sexual debut age, practicing anal sex, other sexually transmitted infections or the use of tobacco or alcohol (Tables S1 and S2).

## 4. Discussion

The present study showed that women of childbearing age acquire primary HSV infections in Finland. The HSV seroprevalence (combined for HSV-1 and HSV-2 as given in the methods) was over 50% in our study cohort. HSV seropositivity increased with age among the women, but not among their spouses. These findings are in agreement with previous studies on HSV seroprevalence in developed countries in the 21st century. A previous study reported an HSV seroprevalence of 54.3% among Finnish women giving birth at Turku University Hospital during one month in the year 2000 [13]. The most recent study on pregnant women (average age 28.5 years) in Finland showed that HSV-1 seroprevalence had further declined from 69.5% (in 1992) to 45% (in 2012) (*p* < 0.001) [11]. In Europe, HSV seroprevalence varies widely, for example, HSV-1 and HSV-2 seropositivity were 52% and 13% in Finland [9], 84% and 24% in Bulgaria [9] and 90.4% and 9.3% in Poland [24]. In all studied European countries [9] as well as in the USA [10] the probability for HSV carriage increased with age.

Recent HSV-seroepidemiological studies have shown that an increasing number of adolescents and young adults lack HSV-1 antibodies at sexual debut [9,10,11,25] and an increasing proportion of genital infections, especially among the young, is caused by HSV-1 [2,3,26,27]. In Finland, the prevalence of HSV-seronegative mothers increased from 25.5% (1992) to 48% (2012) (*p* < 0.001) [11], which indicates that these seronegative pregnant women are susceptible for primary HSV infections and at risk of perinatal HSV infection, which can be life-threatening for the infant [13,28,29,30].

In our cohort, 11.4% (15/132) of the women and 12.5% (8/64) of their spouses seroconverted during the FU, which is consistent with the estimated overall annual rate of transmission; 4–5% per annum [6]. Younger age for women increased the probability for HSV seroconversion. These results imply that women at childbearing age acquire primary HSV infections in Finland. In a previous 20-month FU study on seronegative non-pregnant women (aged 18–30 years), in a different population, the primary HSV infection occurred in 5.3% (183/3438) of the participants [26]. The younger (18–22 years) women were more likely to experience HSV-1 seroconversion [26]. However, another Finnish study on pregnant women (median age 30 years) with two serial samplings during the pregnancy showed no cases with primary HSV infection, leading to the assumption that the risk for viral infections during pregnancy cannot be generalized [13].

Our results showed that women were slightly more often HSV seropositive than their spouses (the test detecting both HSV-1 and HSV-2 antibodies). In line with our results, HSV-1 seroprevalence is also slightly more common in women than men in the US [10] and in Northern Europe (4/7 countries) [9,24].

We have previously analyzed the presence of HSV-1 and HSV-2 DNA in oral brush samples in this cohort, as described earlier [21,22]. Of the HSV-seropositive women, 18.5% (31/168), and 20.3% (13/64) of their HSV-seropositive spouses were oral HSV-1 DNA positive with PCR during the FU, but none had tested HSV-2 DNA positive [21,22]. Two of the 15 seroconverted women (13.3%) and none of the seroconverted spouses had oral HSV-1 DNA detectable during the FU. For example, one seroconverted woman had the HSV antibody level of 70 EIU at the 36-month FU visit and HSV-1 DNA was detected at 222 copies/sample at the 24-month FU visit. Her mean HSV copy number values varied from 13 to 772 HSV DNA copies per sample at different follow-up time points [21]. Interestingly, her spouse was also HSV-1 DNA positive at the same FU visit [22]. He was HSV seronegative at baseline, but had a borderline HSV antibody level (9 EIU) at the 36-month FU visit. Furthermore, his oral brush samples at the 2- and 24-month FU visits tested HSV-1 DNA positive with 16 and 15 DNA copies, respectively.

We can speculate that the HSV-1 shedding among the HSV-1 carriers is even more common, as shedding of the HSV is likely to happen between the FU visits. Furthermore, HSV shedding would be easier to detect in saliva than in epithelial brush samples alone. Our results agree with previous studies, showing that approximately 20–30% of the HSV-1 seropositive individuals have recurrent intraoral HSV-1 infections 1–4 times annually, especially after stressful events [5]. In addition, oral shedding is mostly subclinical and some 70% of the population shed HSV-1 asymptomatically at least once a month, regardless of the seropositivity [5].

Of the HSV-seronegative women, 2.7% (3/112) and 7.4% (4/54) of their spouses had previously yielded a positive HSV DNA result in their oral sample. Intraoral HSV shedding was also found among seronegative individuals in other studies [5]. Both viral culture and PCR might be more sensitive to detect HSV than immunologic assays based on viral antibody detection. Thus, HSV infection could be more prevalent in the population than indicated by the HSV seroprevalence data alone [5].

In our study, the high frequency (self-reported in the questionnaire with the scale: frequently/sometimes/never) of oral sex was statistically significantly associated with the HSV seropositivity in the male spouses, but not among the women. The history of genital warts was associated with the women’s HSV seropositivity, which might partly indicate the sexual habits of the women. Thus, we can speculate that sexual habits influence the acquisition of HSV infection among young women and men in Finland. Earlier studies showed that HSV seroprevalence was higher among sexual risk behavior groups [6,7] increasing along with the number of sexual partners [10,31]. Early age for first sexual intercourse has been associated with HSV-1 seropositivity among young individuals [32], which was, however, not shown in the present study. The overall small fraction of HSV-2 seropositive individuals in Finland was evident also in our HSV antibody results. The majority of the HSV-2 seropositive participants harbored the HSV-2 antibodies already upon entry to our study. We found no HSV-2 DNA in the oral samples of the women or the men in our earlier studies [21,22], which supports the previous observations of HSV-2 lesions being rare in the orofacial area [33].

In conclusion, the HSV seroprevalence was nearly 50% among healthy, young Finnish parents close to the birth of their offspring. Oral HSV-1 shedding is common among HSV-seropositive individuals and contributes to the potential risk of transmission. Increased frequency of oral sex might increase the risk for HSV infection.

## Figures and Tables

**Table 1:** Herpes simplex virus (HSV) serology of the women and their spouses at baseline and at the end of FU.

	HSV * Serostatus			
	Women	Spouses	Total	*p*-Value **
Number of subjects	285	120	405	
Seropositive at baseline (%)	153 (53.7%)	56 (46.7%)	209 (51.6%)	0.198
Median EIU *** value at baseline (range)	71.5 (2–119)	84 (2–114)	74 (2–119)	**0.0017**
Total number of follow-up samples for HSV testing ****	132	64	196	
Seroconverted (%)	15/132 (11.4%)	8/64 (12.5%)	23/196 (11.7%)	0.823
Median EIU value of the seroconverted (range)	70 (10–104)	16 (10–58)	37 (10–104)	**0.0007**
Samples remaining borderline ***** (%)	5/285 (1.8%)	2/120 (1.7%)	7/405 (1.7%)	0.944
Total seropositivity ****** (%)	168 (58.9%)	64 (53.3%)	232 (57.3%)	0.298
		HSV-2 serostatus *******		
Total HSV-2 seropositivity (%)	35/285 (12.3%)	9/120 (7.5%)	44/405 (10.9%)	
HSV-2 seropositivity among HSV seropositive subjects (%)	35/168 (20.8%)	9/64 (14.1%)	44/232 (19.0%)	
Seroconverted subjects with HSV-2 (%)	4/15 (26.7%)	1/8 (12.5%)	5/23 (21.7%)	

* HSV refers collectively to HSV-1 and HSV-2. ** *p*-value indicates the statistical difference between the mean values of the women and their male spouses separately for each variable at every row. Statistically significant *p*-values are bolded. *** Enzyme immune assay (EIA) unit describes here the HSV-1/HSV-2 IgG antibody levels in sera. Median EIA unit values (EIU) have been calculated for the HSV-seropositive individuals. **** The last follow-up serum samples available were also analyzed for those individuals who were HSV seronegative or had borderline results at baseline. At baseline three women had borderline HSV results. The median FU-time for the women was 57.3 (range 6.7–94.5) months and for the spouses 47.8 (range 5.0–91.5) months. ***** Borderline samples were the serum samples with the HSV EIU between 5 and 9. These individuals were HSV seronegative or borderline at baseline and remained as borderline when additionally tested using the last follow-up sample. ****** Total seropositivity includes the number of individuals who were HSV seropositive at baseline and those who seroconverted during the follow-up. ******* HSV-2 type-specific antibody determination was carried out on HSV type-common antibody-positive participants.

**Table 2:** Herpes simplex virus (HSV *) seropositivity among the couples.

HSV-Seropositive Women ** and Their Spouses		HSV-Seropositive Men *** and Their Spouses	
Seropositive Women with Seronegative Spouses	Seropositive Women with Seropositive Spouses	Seropositive Men with Seronegative Spouses	Seropositive Men with Seropositive Spouses
31	47 ****	17	47
39.7% (31/78)	60.3% (47/78)	26.6% (17/64)	73.4% (47/64)

* HSV refers collectively to HSV-1 and HSV-2. ** 58.9% (168/285) women were HSV-seropositive during 6-year follow-up. In total, serum samples were available from 78 spouses of the HSV-seropositive women. *** 120 male spouses, of whom 64 (53.3%) were HSV seropositive during the FU. The HSV statuses of all their female spouses are given. **** Based on the women’s HSV seropositivity, both partners were seropositive in 60.3% (47/78) of the cases. However, of all the couples available in the study (*n* = 120), in 39.2% (47/120) of the cases woman and spouse were both HSV seropositive.

## Supplementary Materials

The following supporting information can be downloaded at: https://www.mdpi.com/article/10.3390/microorganisms10081506/s1, Table S1: Key demographic characteristics of the 285 women stratified according to herpes simplex virus (HSV) serology; Table S2: Key demographic characteristics of the 120 male spouses* according to their herpes simplex virus (HSV) serology.
