# Supplementary material for: Herpes Simplex Virus Seroprevalence among Pregnant Finnish Women and Their Spouses—A Six-Year Follow-Up Cohort Study
Source: Microorganisms. 2022 Jul 26;10(8):1506. doi: 10.3390/microorganisms10081506 (PMC9331543; doi:10.3390/microorganisms10081506)
Supplement: Supplementary file 1 [file microorganisms-10-01506-s001.zip › microorganisms-1812548-supplementary.pdf]

microorganisms

MDPI

Article

Herpes simplex virus seroprevalence among pregnant Finnish women and their spouses – a six-year follow-up cohort study

Johanna Laakso[1,2]*, Tytti Vuorinen[3,4], Jaana Rautava[1,5,6], Katja Kero[7], Stina Syrjänen[1,8] and Veijo Hukkanen[3]

[1] Department of Oral Pathology and Radiology, Institute of Dentistry, and Medicity Research Laboratory, Faculty of Medicine, University of Turku, Turku, Finland
[2] Finnish Doctoral Programme in Oral Sciences, University of Turku, Finland
[3] Institute of Biomedicine, University of Turku, Turku, Finland
[4] Department of Clinical Virology, Turku University Hospital, Turku, Finland
[5] Department of Oral and Maxillofacial Diseases, Clinicum, Faculty of Medicine, University of Helsinki and Helsinki University Hospital, Helsinki, Finland
[6] Department of Pathology, Medicum, Faculty of Medicine, University of Helsinki and HUS Diagnostic Center, HUSLAB, Helsinki University Hospital, Helsinki, Finland
[7] Department of Obstetrics and Gynecology, Turku University Hospital, Turku, Finland and University of Turku, Turku, Finland
[8] Department of Pathology, Turku University Hospital, Turku, Finland
* Correspondence: Department of Oral Pathology, Institute of Dentistry, University of Turku, Lemminkäisenkatu 2, FIN-20520 Turku, Finland. Tel.: +358 45 1386161. E-mail address: johanna.k.laakso@utu.fi

Supplementary Materials

Table S1. Key demographic characteristics of the 285 women stratified according to Herpes simplex virus (HSV) serology.

Variables	Total n* (%)	HSV seropositive n (%)	HSV seronegative n (%)	Seroconverted n (%)	Borderline n (%)	p**
Age in years	285	153	112	15	5	0.48
15-19	5 (1.8%)	2 (1.3%)	3 (2.7%)	0	0	
20-24	100 (35.1%)	45 (29.4%)	45 (40.2%)	9 (60.0%)	1 (20.0%)	
25-29	159 (55.8%)	91 (59.5%)	58 (51.8%)	6 (40.0%)	4 (80.0%)	
30-39	21 (7.4%)	15 (9.8%)	6 (5.4%)	0	0	
Mean age ±SD	25.5±3.2	26.0±3.3	25.0±3.0	23.7±2.0	27.0±2.3	**0.006**
Age of all excluding seroconverted (n)	270			15		
Mean age ±SD	25.63±3.2			23.7±2.0 (n=15)		**0.023**
Education	268	146	104	14	4	0.60
Primary school	22 (8.2%)	11 (7.5%)	8 (7.7%)	3 (21.4%)	0	
Technical school	71 (26.5%)	39 (26.7%)	28 (26.9%)	3 (21.4%)	1 (25.0%)	
High school	50 (18.7%)	25 (17.1%)	22 (21.1%)	3 (21.4%)	0	
Post-secondary level	85 (31.7%)	51 (34.9%)	31 (29.8%)	2 (14.3%)	1 (25.0%)	
University	40 (14.9%)	20 (13.7%)	15 (14.4%)	3 (21.4%)	2 (50.0%)	
Allergy	265	144	103	14	4	0.76
No	150 (56.6%)	78 (54.2%)	60 (58.3%)	9 (64.3%)	3 (75.0%)	
Yes	115 (43.4%)	66 (45.8%)	43 (41.7%)	5 (35.7%)	1 (25.0%)	
Alcohol	267	146	103	14	4	0.67
Never	28 (10.5%)	16 (11.0%)	12 (11.7%)	0	0	
Occasionally	239 (89.5%)	130 (89.0%)	91 (88.3%)	14 (100%)	4 (100%)	
Smoking (cigarettes/day)	268	146	104	14	4	0.94
None	134 (50.0%)	71 (48.6%)	56 (53.8%)	5 (35.7%)	2 (50.0%)	
1-20	129 (48.1%)	72 (49.3%)	46 (44.2%)	9 (64.3%)	2 (50.0%)	
>20	5 (1.9%)	3 (2.1%)	2 (1.9%)	0	0	
Age at onset of smoking	124	70	44	8	2	0.22
10-17 years	106 (85.5%)	60 (85.7%)	38 (86.4%)	7 (87.5%)	1 (50.0%)	
≥18 years	18 (14.5%)	10 (14.3%)	6 (13.6%)	1 (12.5%)	1 (50.0%)	
Mean pack years ±SD	5.06±4.1	5.35±4.2	4.98±4.2	2.94±2.1	5.50±7.1	0.48

Mean menarche age (total n)	12.3 (259)	12.4 (140)	12.1 (101)	13.1 (14)	13.3 (4)	0.29
Mean number of births (total n)	1.29 (268)	1.28 (146)	1.32 (104)	1.21 (14)	1.00 (4)	0.68
Mean number of miscarriages (total n)	0.17 (260)	0.16 (141)	0.20 (101)	0.21 (14)	0	0.77
Mean number of abortion (total n)	0.18 (260)	0.21 (141)	0.14 (101)	0.21 (14)	0.25 (4)	0.60
Sexual debut age	268	146	104	14	4	0.37
<13 years	7 (2.6%)	3 (2.1%)	3 (2.9%)	1 (7.1%)	0	
14-16 years	152 (56.7%)	88 (60.3%)	54 (51.9%)	7 (50.0%)	3 (75.0%)	
17-19 years	97 (36.2%)	48 (32.9%)	44 (42.3%)	4 (28.6%)	1 (25.0%)	
>20 years	12 (4.5%)	7 (4.8%)	3 (2.9%)	2 (14.3%)	0	
Number of sexual partners	267	145	104	14	4	0.18
0-2	66 (24.7%)	31 (21.4%)	31 (29.8%)	3 (21.4%)	1 (25.0%)	
3-5	87 (32.6%)	43 (29.7%)	38 (36.5%)	5 (35.7%)	1 (25.0%)	
6-10	60 (22.5%)	40 (27.6%)	14 (13.5%)	5 (35.7%)	1 (25.0%)	
>10	54 (20.2%)	31 (21.4%)	21 (20.2%)	1 (7.1%)	1 (25.0%)	
Sexual intercourse (n/month)	268	146	104	14	4	0.57
0-1	7 (2.6%)	4 (2.7%)	2 (1.9%)	1 (7.1%)	0	
2-4	85 (31.7%)	53 (36.3%)	28 (26.9%)	3 (21.4%)	1 (25.0%)	
5-10	144 (53.7%)	72 (49.3%)	61 (58.7%)	9 (64.3%)	2 (50.0%)	
>10	31 (11.6%)	17 (11.6%)	12 (11.5%)	1 (7.1%)	1 (25.0%)	
Oral sex	268	146	104	14	4	0.81
Never	56 (20.9%)	28 (19.2%)	23 (22.1%)	4 (28.6%)	1 (25.0%)	
Occasionally	179 (66.8%)	101 (69.2%)	67 (64.4%)	9 (64.3%)	2 (50.0%)	
Frequently	33 (12.3%)	17 (11.6%)	14 (13.5%)	1 (7.1%)	1 (25.0%)	
Anal sex	268	146	104	14	4	0.46
Never	217 (81.0%)	115 (78.8%)	88 (84.6%)	12 (85.7%)	2 (50.0%)	
Occasionally	48 (17.9%)	29 (19.9%)	15 (14.4%)	2 (14.3%)	2 (50.0%)	
Frequently	3 (1.1%)	2 (1.4%)	1 (1.0%)	0	0	
History of sexually transmitted infections	285	153	112	15	5	0.86
None	229 (80.4%)	119 (77.8%)	93 (83.0%)	13 (86.7%)	4 (80.0%)	
Chlamydia	29 (10.2%)	16 (10.5%)	11 (9.8%)	1 (6.7%)	1 (20.0%)	
Genital herpes	10 (3.5%)	8 (5.2%)	2 (1.8%)	0	0	
Multiple:	17 (6.0%)	10 (6.5%)	6 (5.4%)	1 (6.7%)	0	0.72
Genital herpes and other STD	7	5	2	0	0	
Condyloma and other STD	15	9	6	0	0	
History of genital warts	264	144	102	14	4	**0.006**
No	190 (72.0%)	92 (63.9%)	84 (82.4%)	10 (71.4%)	4 (100%)	
Yes	74 (28.0%)	52 (36.1%)	18 (17.6%)	4 (28.6%)	0	
Oral contraceptives	268	146	104	14	4	0.11
Never	23 (8.6%)	11 (7.5%)	8 (7.7%)	4 (28.6%)	0	
Yes	245 (91.4%)	135 (92.5%)	96 (92.3%)	10 (71.4%)	4 (100%)	
Intrauterine device	271	148	105	14	4	0.08
No	211 (77.9%)	109 (73.6%)	87 (82.9%)	13 (92.9%)	2 (50.0%)	
Yes	60 (22.1%)	39 (26.4%)	18 (17.1%)	1 (7.1%)	2 (50.0%)	

*Serological samples were available from 285 women. However, the total number (n) of women analyzed for each variable varies and is seen at first column for every variable also separately for HSV seropositive, HSV seronegative, borderline and HSV seroconverted at first row of each variable. Percent (%) of women in each group has been calculated separately comparing to the total number of women in each group (seen at first row of each variable and at first cell of each column group). ** P-value for each variable is given in the last column and it describes the statistical difference between the groups of each variable. The statistically significant p-values are bolded.

Table S2. Key demographic characteristics of the 120 male spouses* according to their Herpes simplex virus (HSV) serology.

Variables	Total n* (%)	HSV seropositive n(%)	HSV seronegative n(%)	Seroconverted n(%)	Borderline n(%)	p
Age (years)	119	55	54	8	2	0.68
15-19	1 (0.8%)	0	1 (1.9%)	0	0	
20-24	17 (14.3%)	6 (10.9%)	9 (16.7%)	2 (25.0%)	0	
25-29	59 (49.6%)	23 (41.8%)	29 (53.7%)	5 (62.5%)	2 (100%)	
30-44	42 (35.3%)	26 (47.3%)	15 (27.8%)	1 (12.5%)	0	
Mean age ±SD	28.7±5.1	29.6±5.1	28.2±5.2	26.5±3.8	27.5±2.1	0.28
Age of all excluding seroconverted (n)	111			8		0.20
Mean age ±SD	28.9±5.1			26.5±3.8		
Education	113	51	52	8	2	0.72
Primary school	8 (7.1%)	3 (5.9%)	3 (5.8%)	1 (12.5%)	1 (50.0%)	
Technical school	48 (42.5%)	24 (47.1%)	21 (40.4%)	2 (25.0%)	1 (50.0%)	
High school	10 (8.8%)	3 (5.9%)	6 (11.5%)	1 (12.5%)	0	
Post-secondary level	30 (26.5%)	14 (27.5%)	13 (25.0%)	3 (37.5%)	0	
University	17 (15.0%)	7 (13.7%)	9 (17.3%)	1 (12.5%)	0	
Allergy	110	51	50	7	2	0.13
No	65 (59.1%)	34 (66.7%)	26 (52.0%)	5 (71.4%)	0	
Yes	45 (40.9%)	17 (33.3%)	24 (48.0%)	2 (28.6%)	2 (100%)	
Alcohol	112	52	51	7	2	0.48
Never	2 (1.8%)	0	2 (3.9%)	0	0	
Occasionally	110 (98.2%)	52 (100%)	49 (96.1%)	7 (100%)	2 (100%)	
Smoking (cigarettes/day)	111	52	50	7	2	0.53
None	71 (64.0%)	31 (59.6%)	33 (66.0%)	6 (85.7%)	1 (50.0%)	
1-20	31 (27.9%)	17 (32.7%)	13 (26.0%)	0	1 (50.0%)	
>20	9 (8.1%)	4 (7.7%)	4 (8.0%)	1 (14.3%)	0	
Age at onset of smoking	44	22	19	2	1	0.76
10-17 years	38 (86.4%)	18 (81.8%)	17 (89.5%)	2 (100%)	1 (100%)	
≥18 years	6 (13.6%)	4 (18.2%)	2 (10.5%)	0	0	
Mean pack years ±SD	10.46±9.5	9.60±5.6	11.43±13.1	11.75±6.7	elo.25	0.93
Sexual debut age	113	52	51	8	2	0.11
<13 years	3 (2.7%)	2 (3.8%)	0	1 (12.5%)	0	
14-16 years	47 (41.6%)	26 (50.0%)	17 (33.3%)	2 (25.0%)	2 (100%)	
17-19 years	51 (45.1%)	18 (34.6%)	28 (54.9%)	5 (62.5%)	0	
>20 years	12 (10.6%)	6 (11.5%)	6 (11.8%)	0	0	
Number of sexual partners	113	52	51	8	2	0.31
0-2	16 (14.2%)	5 (9.6%)	8 (15.7%)	2 (25.0%)	1 (50.0%)	
3-5	24 (21.2%)	10 (19.2%)	13 (25.5%)	0	1 (50.0%)	
6-10	27 (23.9%)	14 (26.9%)	12 (23.5%)	1 (12.5%)	0	
>10	46 (40.7%)	23 (44.2%)	18 (35.3%)	5 (62.5%)	0	
Sexual intercourse (n/month)	110	51	49	8	2	0.18
0-1	1 (0.9%)	1 (2.0%)	0	0	0	
2-4	39 (35.5%)	17 (33.3%)	20 (40.8%)	1 (12.5%)	1 (50.0%)	
5-10	54 (49.1%)	25 (49.0%)	25 (51.0%)	3 (37.5%)	1 (50.0%)	
>10	16 (14.5%)	8 (15.7%)	4 (8.2%)	4 (50.0%)	0	
Oral sex (n)	112	52	50	8	2	**0.033**
Never	13 (11.6%)	4 (7.7%)	9 (18.0%)	0	0	
Occasionally	70 (62.5%)	36 (69.2%)	31 (62.0%)	3 (37.5%)	0	
Frequently	29 (25.9%)	12 (23.1%)	10 (20.0%)	5 (62.5%)	2 (100%)	
Anal sex (n)	112	52	50	8	2	0.16
Never	88 (78.6%)	40 (76.9%)	42 (84.0%)	4 (50.0%)	2 (100%)	
Occasionally	24 (21.4%)	12 (23.1%)	8 (16.0%)	4 (50.0%)	0	
History of sexually transmitted infections	45	20	21	3	1	0.80
None	21 (46.7%)	8 (40.0%)	11 (52.4%)	1 (33.3%)	1 (100%)	
Chlamydia	15 (33.3%)	6 (30.0%)	7 (33.3%)	2 (66.7%)	0	
Genital herpes	3 (6.7%)	2 (10.0%)	1 (4.8%)	0	0	
Multiple:	5 (11.1%)	4 (20.0%)	1 (4.8%)	0	0	1.0
Genital herpes and other STD	2	2	0	0	0	

Condyloma and other STD	3	2	1	0	0	
History of genital warts	105	48	48	7	2	0.94
No	88 (83.8%)	39 (81.3%)	41 (85.4%)	6 (85.7%)	2 (100%)	
Yes	17 (16.2%)	9 (18.8%)	7 (14.6%)	1 (14.3%)	0	

*Serological samples were available from 120 male spouses. However, the total number (n) of men analyzed for each variable varies and is given for every variable also separately for HSV seropositive, HSV seronegative, borderline and HSV seroconverted men. Percentage (%) of the men in each group has been calculated separately comparing to the total number of men in each group (seen at first row of each variable and at first cell of each column group). **P-value for each variable is given in the last column and it describes the statistical difference between groups of each variable at the precise row. The statistically significant p-values are bolded.
